# The need to standardize use of the newly deceased in medical trainings

**DOI:** 10.6061/clinics/2020/e2391

**Published:** 2020-11-10

**Authors:** Fábio Roberto Cabar, Daniele Costa Rachid Lacerda, Gabriela Thomé Souza de Freitas, Maria Luiza Gorga

**Affiliations:** I Faculdade de Medicina (FMUSP), Universidade de Sao Paulo, Sao Paulo, SP, BR; II Faculdade de Medicina, Universidade Federal de Juiz de Fora, Juiz de Fora, MG, BR; III Faculdade das Americas, Sao Paulo, SP, BR; IV Hospital das Clinicas (HCFMUSP), Faculdade de Medicina, Universidade de Sao Paulo, Sao Paulo, SP, BR

**Keywords:** Cadaver, Principle-Based Ethics, Control Social Formal, Manikins, Health Behavior

## Abstract

**OBJECTIVES:**

The present study aimed to identify the characteristics of use of the deceased in invasive training and the bioethical principles that govern this practice. In this context, it has become imperative to deduce which professional skills are critical to develop.

**METHODS:**

A prospective study investigated a cadaver’s use in medical (and related) schools through a questionnaire, which was made available for 48 hours on social networks (Facebook and LinkedIn) to groups of doctors and medical students using a communication app (WhatsApp). The inclusion criteria were being a medical student or a doctor. Cases in which the answers to the questionnaire were inadequate, or when the student had reason to withdraw, were excluded. Each participant could only answer the questionnaire once, and could not modify the responses after submitting it.

**RESULTS:**

A disproportionate relationship was found regarding the replacement of the newly deceased by other means (such as dummies and simulators). This outcome suggests that there is no substitution, concomitant with the importance of a prior request for consent from the patient and/or subsequent consent from family members.

**CONCLUSION:**

According to the findings, the significance of—and need for—training is undeniable. Hence, it is urgent to normalize the practice and definition of the ethical limitations of medical conduct.

## INTRODUCTION

In hospitals, performing delicate procedures is routinely necessary to care for patients, mostly in difficult situations where time is of the essence. Any mistake can cost patients their lives, and studies indicate that preventable medical errors are the third leading cause of death in the US (1), a reality that is also of concern in Brazil (2). This implies the need for strict, accurate training.

To properly practice medicine, and to fully realize the bioethical principles of beneficence and non-maleficence that govern medical conduct, those who practice medical activities should undergo a rigorous qualification process and receive continuous training. This way, personnel learn in advance the best way to perform complex procedures and to improve the techniques they employ, including invasive and/or preoperative procedures.

In this realm of knowledge, during all professional education (whether during one’s undergraduate career, medical residency, or specialization), trainings and studies on preserved cadavers, simulators, and dummies—as well as on patients—are performed in university hospitals around the world.

The present study describes another means of training: the use of the so-called recently dead; that is, that newly deceased patient, whose body is utilized to train medical staff for certain invasive procedures. This practice is often performed in secret, without formal authorization (from patients or their guardians), and is fundamental to medical training (3).

This practice is justified because if training on live individuals carries risks, those already deceased cannot suffer any harm. Moreover, the human body (with all its peculiarities) is impossible to faithfully reproduce in simulations, and training situations in fully controlled environments do not instill in students the relationship between fear and stress control; thus, practice on a cadaver is essential to increase not only a doctor’s ability, but also to boosting their self-confidence (4-7).

If such training can help professionals to fully develop their skills and master techniques to enhance care for individuals, the dilemma is whether this facet of medical education (as it is performed today) is ethical (8).

Despite the above being a common theme in medical practice, recent research has observed that the general population knows next to nothing of the matter. People whose relatives have recently died do not imagine that this practice may be occurring; this is clearly not in line with the precepts of medical ethics.

## MATERIAL AND METHODS

Given the scarcity of material in the national literature, we opted for a prospective study on a cadaver’s use in medical (and related schools) through a questionnaire, which we made available for exactly 48 hours on social networks (Facebook and LinkedIn) to groups of doctors and medical students using a communication app (WhatsApp). The inclusion criteria were that the participant should be a medical student or a doctor. We excluded cases for which the answers to the questionnaire were inadequate, or when the student had reason to withdraw. Each participant could only answer the questionnaire once, and could not modify the responses after submitting it.

**Figure 1:**
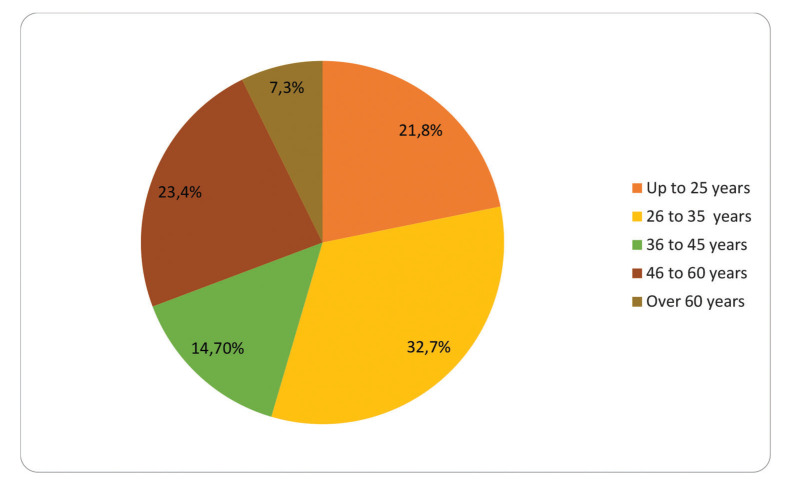
Age distribution.

We present the data descriptively. We obtained 503 answers within 48 hours. The majority (53.9%) of the respondents were female, 430 (85.5%) were doctors, and the rest (73 respondents) were medical students from several different years. Figure 1 depicts the age distribution.

## RESULTS

Of the 503 responses obtained, 353 (70.2%) participants stated they had performed or received training on newly deceased individuals and cited several procedures that were carried out. Of those who said they had already taken part in such training, 297 had done so as undergraduate students, 87 as doctors (whether they were residents or not), and 39 as medical teachers. The most common trainings were endotracheal intubation (209 responses), central venous catheterization (115 responses), chest drainage (84 responses), and tracheostomy (51 responses). Other procedures such as gynecological surgeries, appendectomies, other gastrointestinal surgeries, and endonasal surgeries were reported as well.

Regarding the importance of the training, 77.5% of the participants understood that it cannot be replaced by other means (such as dummies and/or simulators) with the same quality and reliability.

In terms of those who performed and/or gave the training, 93% affirmed that no prior request for consent had been obtained from the patient, and 86% denied that consent had been sought from family members. Only 14.3% of the respondents said they believed that consent for procedures would be unnecessary, and just 10.5% claimed they had already been denied consent by patients or relatives.

Finally, approximately 86.4% of the respondents believe this type of training is important enough to warrant better regulation from an ethical and legal perspective.

## DISCUSSION

The fact that medical professionals of all ages (from 25 to upwards of 60) have reported that they have already engaged in this kind of training indicates that the practice has been widespread in medical education for many years.

The answers to the questionnaire signal that numerous procedures are practiced/taught using the recently deceased, from simpler medical ones (such as endotracheal intubation and venous access catheterization) to vital surgeries (such as appendectomies and colectomies). This fact reinforces the importance of using such models in medical learning. Some responders have received training at more than one point in their lives (*e.g.*, as students, doctors in training, or when they are already teachers). Likewise, some participants have performed more than one type of procedure during their medical education.

The overwhelming majority stated that training on the recently deceased cannot be replaced by any other means of equal quality and reliability.

This impression confirms studies that show that—except for using live humans—no other training model has the same advantages and particularities of training using fresh cadavers. The primary advantage of using new cadavers is the similarity of the actual situation with a model that reflects true human anatomy, while the main disadvantages of these models would be the need to obtain family consent, the limited time for use, the potential risk of disease transmission, and the ethical positions of some students and doctors. Otherwise, the use of mannequins and other artificial models would bring benefits such as training on various procedures without the need to obtain consent, the absence of ethical issues, the possibility of using the same model various times, and immediate and continuous availability. Key drawbacks include limited realism, cost, and the need for maintenance (9).

Live animal models have also been employed in medical education for many years (10,11). However, these require high maintenance costs related to animal care and ethical requirements, so their use might not be helpful for basic procedures. On the other hand, several models use animal segments to train people on resuscitation and chest drainage skills, for example, with the advantages of lower costs and easy reproduction, making them attractive models in the early stages of medical education, particularly in centers where resources are restricted (12,13). However, there is no denying that the use of animal models does not bring about the actual anatomical difficulties related to procedures in humans, such as bruising, rib fractures, obesity, and emergency situations.

A small number of respondents reported that they sought (or tried to seek) consent from patients or relatives to perform the abovementioned procedures. Contrary to this fact, the vast majority asserted that such consent should be sought, and over 90% of the participants said they never saw consent being denied when requested. This demonstrates that despite recognizing the importance of consent and the ease of obtaining it, such an attitude is not usually adopted in practice. It may be strange to ask for consent from the patient himself/herself; yet this would not be impossible (or even strange) if, at the time of hospitalization, the patient was asked if procedures for medical training and improvement could be performed if the course of treatment were not favorable.

In Brazil, the only major study on this issue, covering practices from 1977 to 2007, concluded that prior or later consent from the family should be required, and that the practice should be restricted to procedures that are non-mutilating, under the strict supervision of professors, and preferably after first training with simulators (14).

A study conducted in the US found similar results. Training of this type was performed by up to 63% of the medical professionals who participated (depending on the type of hospital unit), and consent was sought in only 10% of cases (8).

Therefore, the subject is relevant and current, having ethical (and possibly criminal) implications, since the current legislation (Law 8.501/1992)—which provides for the use of unclaimed corpses for study purposes or scientific research—does not apply to the newly deceased. In fact, there are no legal rules governing the practice.

In the absence of specific regulations, it would be possible to frame the practice as a crime against respect for the dead, such as destruction or vilification of a corpse. The nature of such offenses is undoubtedly moral. In the case of scientific and educational studies, such moral guardianship should not be applied, since the act performed is intended to improve professional skills and to ensure that patients in the future can be helped. Hence, in the name of allegedly protecting the morale of the dead, they turn against the living, who would be deprived of well-trained professionals to adequately serve them.

Illustratively, a single case from 1976, tried by the Federal Supreme Court (HC 54.486), found that criminal action brought against two medical students and a professor, then denounced for corpse vilification, should be extinguished due to the clear scientific rationale underlying their conduct. In this specific case, a corpse that was in the morgue of the Faculty awaiting an autopsy was used without any consent or donation of the body to science; the procedures performed included ocular enucleation.

From an ethical angle, the Code of Medical Ethics does not contain any rules on the use of a corpse as an object of teaching or learning. Likewise, no resolution or opinion of the Federal Council (or Regional Councils) of Medicine has been identified on the issue. On the other hand, the Medical Student Code, Article 13, states that it is the student’s duty to “respect the corpse, in whole or in part, including any anatomical parts, as well as anatomical models used for learning purposes.”

The importance of this kind of training is unquestionable for all future benefits related to the performance of professionals who are adequately prepared to act in emergency circumstances. Moreover, the value protected by a greater efficiency (public health) prevails over any moral objection.

However, the ethical guidelines of the medical profession must be respected. Otherwise, there would be distrust in the classroom, and moral principles would be degraded.

Moore (14) argues that the matter should not be about the need for such training (considered essential), but rather about its standardization and obtaining consent from family members. We go so far as to maintain that all newly deceased in emergency rooms could be used for training. Exceptions should include those whose cause of death is not certain, or who were victims of violent deaths. In such cases, the body should be sent, intact, to the Death Verification Service or the Forensic Medical Institute. This is reasonable from the standpoint of the equal treatment of corpses, preventing certain individuals, when dead, from being given preference for being used for training medical personnel.

Regarding consent, José Marques Filho—one of the only people to deal with the issue at national level—questions whether requesting consent from family members would cause unnecessary (and even inhuman) mental anguish (15). Therefore, we should consider the non-maleficence principle in guiding doctors’ behavior, including in relation to family members.

At this point, one resolution would involve establishing transparent policies on the subject, to be openly implemented in university hospitals and medical residency centers, so that both patients and family members are informed about the matter in advance and could choose not to give consent.

Such a policy would avoid emotional distress for family members and professionals in pursuit of later consent, and would eliminate the need for—in the eagerness to act on the body before it loses its dynamic characteristics—procedures to be carried out in secret and/or in clear violation of family members’ wishes.

In agreement with our findings, previous studies (from other countries) indicate that such a policy would engender an expressive amount of consent (16) so that this practice could become widespread. After all, it is easier to perform procedures openly (even if only on those who have consented to it) than to do so surreptitiously. As such, the quantity of cadavers available for trainings would certainly be larger.

Finally, there is the need for trust and honesty in the patient-medical relationship; full disclosure is a critical pillar of this relationship (17). The professionals’ unique safeguarding of interests cannot be allowed to take precedence over the beneficence that should guide their conduct regarding patients.

## CONCLUSION

Given the above and the outcomes of the research—according to which, the great majority of the participants (86.4%) corroborated the benefits of this type of training, and that its importance justifies its regulation—we believe that the Federal Council of Medicine, within its role as a federal autarchy with normative authority, must set forth specific rules.

In addition, hospitals that have students and residents on their staff must develop internal regulations using their own codes of conduct, consent forms, and the formalization of this practice (or opting out of it). The creation of such rules and the flow of behavior could occur even before the Federal Council of Medicine takes action, thus preserving both the consolidation of this crucial practice and the bioethical principles that govern it.

## AUTHOR CONTRIBUTIONS

Cabar FR, contributed to the design of the revision and wrote the manuscript. Lacerda DC wrote the manuscript. de Freitas GT and Gorga ML revised and wrote the manuscript.
